# Validation of ultrasound velocimetry and computational fluid dynamics for flow assessment in femoral artery stenotic disease

**DOI:** 10.1117/1.JMI.11.3.037001

**Published:** 2024-05-16

**Authors:** Lennart van de Velde, Majorie van Helvert, Stefan Engelhard, Ashkan Ghanbarzadeh-Dagheyan, Hadi Mirgolbabaee, Jason Voorneveld, Guillaume Lajoinie, Michel Versluis, Michel M. P. J. Reijnen, Erik Groot Jebbink

**Affiliations:** a University of Twente, TechMed Centre, Multi-Modality Medical Imaging, Enschede, The Netherlands; b University of Twente, TechMed Centre, Physics of Fluids, Enschede, The Netherlands; c Rijnstate Hospital, Department of Surgery, Arnhem, The Netherlands; d Erasmus MC, Department of Cardiology, Thorax Biomedical Engineering, Rotterdam, The Netherlands

**Keywords:** blood flow imaging, computational fluid dynamics, echo-particle image velocimetry, peripheral arterial disease, stenotic blood flow, ultrafast ultrasound imaging

## Abstract

**Purpose:**

To investigate the accuracy of high-framerate echo particle image velocimetry (ePIV) and computational fluid dynamics (CFD) for determining velocity vectors in femoral bifurcation models through comparison with optical particle image velocimetry (oPIV).

**Approach:**

Separate femoral bifurcation models were built for oPIV and ePIV measurements of a non-stenosed (control) and a 75%-area stenosed common femoral artery. A flow loop was used to create triphasic pulsatile flow. In-plane velocity vectors were measured with oPIV and ePIV. Flow was simulated with CFD using boundary conditions from ePIV and additional duplex-ultrasound (DUS) measurements. Mean differences and 95%-limits of agreement (1.96*SD) of the velocity magnitudes in space and time were compared, and the similarity of vector complexity (VC) and time-averaged wall shear stress (TAWSS) was assessed.

**Results:**

Similar flow features were observed between modalities with velocities up to 110 and 330  cm/s in the control and the stenosed model, respectively. Relative to oPIV, ePIV and CFD-ePIV showed negligible mean differences in velocity (<3  cm/s), with limits of agreement of ±25  cm/s (control) and ±34  cm/s (stenosed). CFD-DUS overestimated velocities with limits of agreements of 13±40 and 16.1±55  cm/s for the control and stenosed model, respectively. VC showed good agreement, whereas TAWSS showed similar trends but with higher values for ePIV, CFD-DUS, and CFD-ePIV compared to oPIV.

**Conclusions:**

EPIV and CFD-ePIV can accurately measure complex flow features in the femoral bifurcation and around a stenosis. CFD-DUS showed larger deviations in velocities making it a less robust technique for hemodynamical assessment. The applied ePIV and CFD techniques enable two- and three-dimensional assessment of local hemodynamics with high spatiotemporal resolution and thereby overcome key limitations of current clinical modalities making them an attractive and cost-effective alternative for hemodynamical assessment in clinical practice.

## 1 Introduction

Peripheral arterial disease (PAD) is a major cardiovascular disease burden with a global prevalence of 10% to 25% in age groups above 65 years,^1^ with the femoral artery as a key site of atherosclerotic manifistations.^2^ It is well known that areas with complex flow structures, such as recirculation zones, induce low and oscillatory wall shear stress (WSS), thereby forming a local risk factor for atherosclerosis.^3^ Flow disturbances induced by the femoral bifurcation, the triphasic flow waveform including a pronounced backflow phase,^4^ and the confined anatomic space of the superficial femoral artery (SFA)^5^ may be the leading causes for the high prevalence of atherosclerotic lesions in this region. The association between adverse flow patterns and the onset and progression of atherosclerosis in patients has so far not been studied in-depth, mainly due to a lack of cost-effective and validated *in-vivo* flow measurement techniques.

The current diagnostic workflow to examine blood flow in PAD patients involves duplex ultrasound (DUS), a combination of conventional B-mode ultrasound and pulsed-wave Doppler measurements. Although DUS is widely available and inexpensive, it only provides a one-dimensional velocity estimate in the axial beam direction, which is prone to overestimation^6^ and often inaccurate in complex flow due to averaging and due to the difficulty of estimating the correct local beam-to-flow angle.^7^^–^^9^

Besides DUS, phase contrast MRI (PC-MRI) can also be used to study time-resolved three-dimensional hemodynamics. However, PC-MRI has limited availability, is expensive, time consuming, and has a low spatiotemporal resolution. Moreover, it averages the phase information over multiple cardiac cycles thereby losing insights in cycle-to-cycle variations.^10^^,^^11^

Multiple innovative approaches, using a variety of imaging modalities, have promising and synergistic potential for blood flow quantification in patients. First of all, several ultrasound techniques, such as multi-directional Doppler imaging and tracking of blood speckle or ultrasound contrast agents (UCA), can be used to measure two-dimensional local hemodynamics.^12^^,^^13^ Advances in plane wave ultrasound imaging improved the acquisition process by enabling a framerate of over 1000 frames per second (fps), thereby vastly overcoming the limited temporal resolution of PC MRI. For complex flows in stenosed vessels, speckle tracking presents a key advantage: it does not suffer from a tradeoff between tracking low and high velocities, in contrast to Doppler-based imaging where the pulse repetition frequency influences its sensitivity to certain velocities due to aliasing. Tracking of UCA, referred to as ultrafast ultrasound particle image velocimetry or echoPIV (ePIV), has been validated *in vitro* against the gold standard, laser optical PIV (oPIV), in various anatomically realistic models, such as the left ventricle^14^ and the carotid bifurcation.^15^ Furthermore, its capability to measure blood flow in stented femoral arteries of patients with PAD has recently been demonstrated.^16^ However, these *in vitro* and *in vivo* studies did not investigate significant transitional flows, i.e., the flows with mixed laminar and turbulent characteristics that could arise downstream of a stenosis. The accuracy of ePIV in stenotic lesions, which comprise large velocity gradients and many small-scale vortices, is thus yet to be demonstrated.

Alternatively, computational fluid dynamics (CFD) modeling in patient-specific geometries can be used to simulate three-dimensional velocity profiles and associated flow parameters.^17^ CFD has shown its benefit across the field of cardiovascular medicine to study complex physiological flow and to calculate different hemodynamical parameters, such as stenotic pressure gradients^18^ and WSS.^19^ For pressure gradients in stenosed coronary arteries, extensive clinical validation has been performed,^20^ but the validation of velocity fields computed by CFD in pulsatile stenotic flows is limited; one study in particular has demonstrated the ability of a spectral-element method for simulating the complex pulsatile transitional flow in a stenosis.^21^

Comparisons of this direct numerical simulation to Reynolds-averaged Navier Stokes (RANS) turbulence models in CFD highlighted the shortcomings of these models when assessing the transitional flow behavior. Most importantly, these RANS models provided erroneous predictions in the onset of turbulence and the relaminarization length.^21^ An under-resolved CFD simulation without an explicit turbulence model or a large eddy simulation model, where the computational cost is reduced by implicitly or explicitly modeling turbulence for only the small, subgrid, length scales (instead of all length scales with RANS models), is likely better suited for simulating stenotic flows in patients.^22^

Second, CFD results critically depend on the applied boundary conditions, which consist of the segmented geometry and the flow conditions enforced on the inlets and outlets. Most validation studies have applied optimal boundary conditions as they were based on the experimental technique that it was compared to.^23^^,^^24^ Thus, CFD accuracy with current clinical techniques is directly limited by the spatial resolution of the applied imaging modality. Flow information for setting the boundary conditions is typically only available from DUS.

Given the higher resolution, absence of angle-dependency and a 2D or 3D blood velocity quantification, ePIV and CFD—or a combination of both—might provide novel insights in local hemodynamics. This provides the ability to assess new parameters of flow disturbances in patients, such as WSS and vector complexity (VC). VC has recently been used to evaluate lesion severity in SFA stenoses and outperformed the conventional velocity ratio obtained with DUS.^25^ As such, ePIV and CFD might improve the diagnosis of PAD and provide a more patient-specific and durable treatment. However, the implementation of ePIV and CFD in clinical practice requires validation in clinically relevant flow conditions. This study investigates the accuracy of ePIV and CFD with ePIV and DUS-derived boundary conditions for determining spatiotemporal velocity vector fields in a non-stenosed and stenosed femoral bifurcation model by comparison to oPIV as a ground-truth reference technique.

## 2 Methods

### 2.1 Femoral Bifurcation Model

**Fig. 1:**
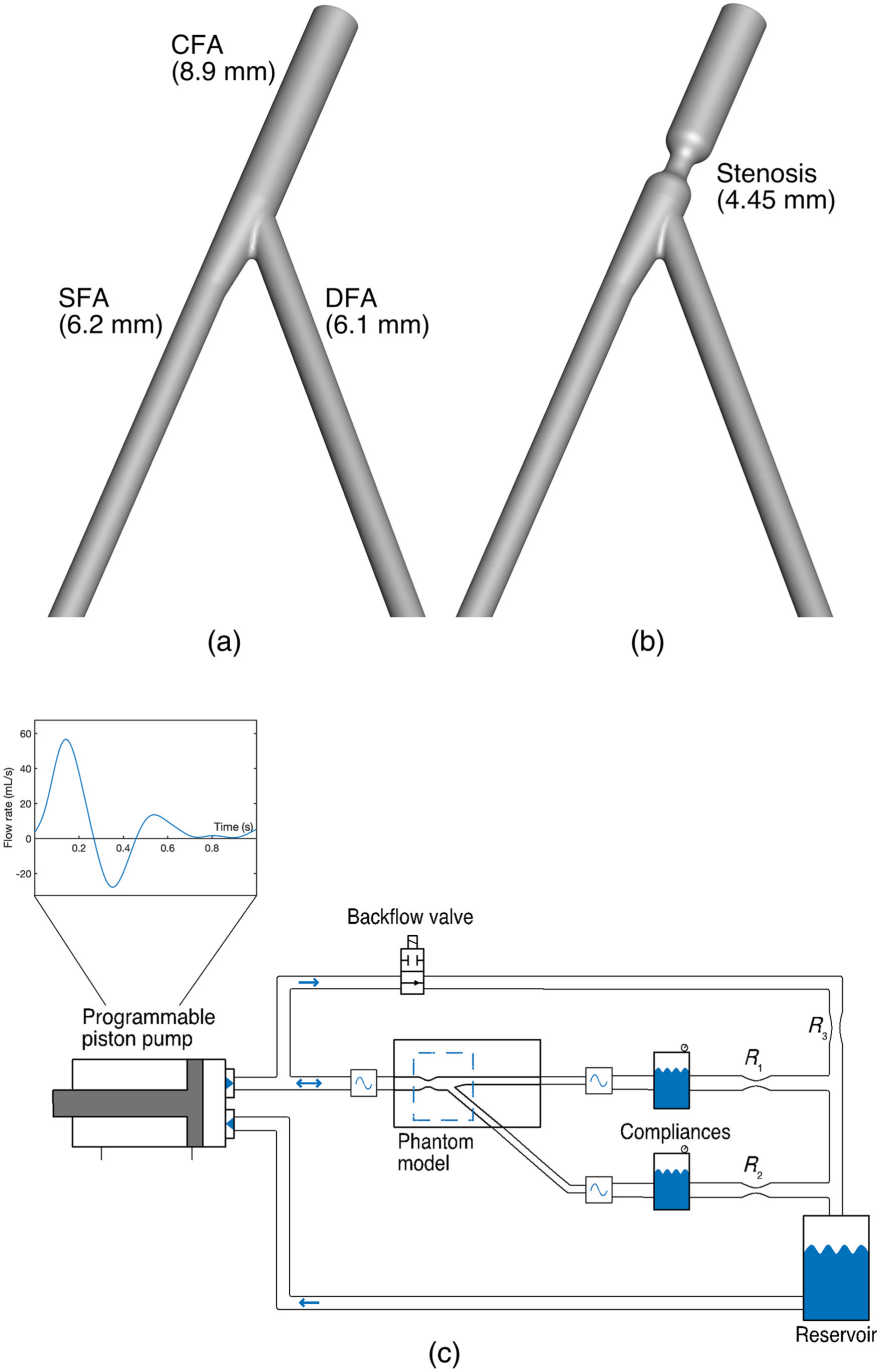
Experimental setup. (a) Control and (b) stenosed geometry used for the flow models. (c) Circulating flow loop. R, resistance.

The geometry of the femoral bifurcation was based on a literature review (Appendix A) and was constructed as follows: common femoral artery (CFA) diameter of 8.9 mm, SFA diameter of 6.2 mm, and deep femoral artery (DFA) diameter of 6.1 mm. To facilitate imaging, the bifurcation was kept in a single plane with a 37.5-deg angle between the SFA and DFA. Two models were made, one without stenosis [control model, Fig. 1(a)] and one with a 75%-area concentric stenosis of the CFA [stenosed model, Fig. 1(b)].

Since no single available material was satisfactorily suited for both oPIV and ePIV, twin models were produced, one for oPIV and one for ePIV. The oPIV models were fabricated using the lost core technique: the geometries were first printed (Ultimaker S5, Ultimaker, Geldermalsen, The Netherlands) with hollow acrylonitrile butadiene styrene (ABS) and, after acetone vapor smoothing, casted in polydimethylsiloxane (PDMS, Sylgard 184, Dow Corning, Midland, Michigan). After curing, the ABS parts were manually extracted. Residual ABS that could not be removed was dissolved by running an acetone solution through the flow lumen. The ePIV models were constructed by 3D-printing the geometries with a flexible resin (Flexible 80A; Form 3, Formlabs, Somerville, Massachusetts) with 50  μm resolution and 1 mm wall thickness. The flexible resin had a speed of sound that matched that of the blood mimicking fluid (BMF) and had low enough attenuation that allowed for UCA visualization and flow quantification.

To assess the geometric similarity of the established lumens in the optical and acoustical twin model, a cone-beam computerized tomography (CT) scan (Artis Pheno, Siemens AG, Berlin, Germany, spatial resolution 0.47 mm) was made. The geometry was segmented with a subpixel-accurate level-set approach. Subsequent analysis of the centerline and inscribed-sphere based radius of the models showed a comparable SFA-DFA angle (oPIV model 37.8 deg; ePIV model 37.4 deg) and deviations in the radius of the CFA and SFA in the order of 0.25 mm (Appendix B).

### 2.2 Experimental Flow Setup

The models were placed in a circulating flow setup, as schematically presented in Fig. 1(c). A triphasic flow profile, derived from DUS measurements in the CFA of seven healthy volunteers (four male, three female; aged 20 to 30 years), was replicated at a frequency of 1 Hz, i.e., 60 beats per minute (Appendix A). A hydraulic piston pump (SuperPump, ViVitro labs, Victoria, Canada) was used to enforce time-varying forward flow. The downstream vasculature of both the SFA and DFA was modeled with a tunable Windkessel model (air chamber) and tunable resistance. An electronically controlled valve was used to reproduce the backflow phase, during which the pump valve is closed and the built-up Windkessel pressure drives flow in retrograde direction through the opened electronic valve (electronically synchronized with the piston pump). Ultrasonic transit time flow sensors in the setup (Sonoflow CO.55, Sonotec, Halle, Germany) were used to establish target mean flow rates by tuning the piston amplitude and resistances. The amplitude of the piston was set such that a flow volume of 9.6 mL was ejected during forward flow, of which 2.3 mL returned during the backflow phase. The SFA and DFA resistances were tuned to enforce a mean 44%:56% flow split (Appendix A). A mixture of 44 w% water, 34.5 w% glycerol, and 21.5 w% urea was used as BMF, which had an optical refractive index matched to PDMS.^26^ Density of the BMF was measured at $1.144\;\mathrm{kg}/m^{3}$ with a density meter (Anton Paar GmbH, Austria) and viscosity at 4.17 mPa.s with an Ubbelohde viscometer (Xylem Analytics, Germany), which comply with the physiological range of blood.

### 2.3 Optical Particle Image Velocimetry

Fluorescent spheres (rhodamine-coated; size, 1 to 20  μm; density, $1190\;\mathrm{kg}/m^{3}$; Dantec Dynamics A/S, Skovlunde, Denmark) were added to the BMF as tracer particles and illuminated with a thin laser sheet (sheet thickness maximum: 0.8 mm, 5-W DPSS laser, 532 nm; Cohlibri, Lightline, Germany) that was passed through the center of the bifurcated flow lumen. A high-speed camera (FASTCAM SA-Z; Photron Inc., West Wycombe, Buckinghamshire, United Kingdom) was positioned perpendicular to the laser sheet and used to capture 10 cardiac cycles of the bifurcation ($55\times 55\;{\mathrm{mm}}^{2}$) at 8000 frames per second (fps).

Data analysis was performed in MATLAB R2022b (The Mathworks, Natick, Massachusetts). The images were first filtered with a moving minimum intensity projection background subtraction (window length = 500 frames) and preprocessed with contrast-limited adaptive histogram equalization. Subsequently, the 2D velocity vector fields were derived through PIV analysis using the PIVlab toolbox, which tracked velocities in a static (Eulerian) grid of rectangular image windows.^27^ Three iterations of block wise normalized cross-correlation were applied with progressive grid refinement. Square interrogation windows of 64, 32, and 16 pixels with 50% overlap were used for the successive iterations, yielding a final isotropic vector resolution of 0.42 mm. Final postprocessing included a 10-ensemble temporal averaging filter, resulting in 400 vector fields per second.

### 2.4 Echo Particle Image Velocimetry

The ePIV measurements were performed with a Vantage 256 ultrasound system connected to a linear transducer (L11-4v; Verasonics, Kirkland, Washington). The printed geometries were encased in a reservoir and submerged in BMF. The reservoir contained an acoustical window to which the transducer was fixed to position the center of the lumen in the elevation focus, at a depth of 15 to 25 mm. As such, the alignment between the oPIV and ePIV measurements was assured. A UCA (Sonovue microbubbles; Bracco Imaging, Milan, Italy) was added to the BMF as tracer particle after which images were captured for 3 s using a three-angled plane wave acquisition scheme (4 MHz center frequency, single-cycle pulse) at 9000 fps. These settings are feasible in the clinical setting as well since the acoustical output is within safety limits. The 3-s acquisition was performed four times to capture a minimum of 10 cardiac cycles. Because of the limited aperture of the transducer (38 mm), two consecutive field of views were acquired to capture the entire region of interest. First, the CFA and proximal bifurcation were imaged. Second, the distal bifurcation, SFA, and DFA were imaged. The fields of view had an overlap of ∼1  cm, which was later used to automatically register the field of views and fuse the velocity vector fields.

The reconstructed images for the different angles were coherently compounded—yielding an effective frame rate of 3000 fps—and filtered by means of singular value decomposition. The lower and higher rank thresholds were semi-automatically found based on the spatial similarity matrix.^28^ Similar to oPIV, the 2D velocity vector fields were calculated through PIV analysis. Here, a modified implementation of PIVlab was employed, as previously used for ePIV in a clinical setting.^16^^,^^29^ For the control model, six iterations with square interrogation windows of 32, 32, 16, 16, 8, and 8 pixels and 75% overlap were used, corresponding to a final isotropic vector grid spacing of 0.22 mm. For the stenosed model, two initial iterations with a square size of 64 pixels were added to capture the higher stenotic velocities. Correlation averaging of 10 frames was applied to increase the signal-to-noise ratio, resulting in 300 vector fields per second. Final postprocessing consisted of a spatial Gaussian filter (3×3 velocity vectors) and a three-ensemble temporal moving average filter.

### 2.5 Doppler Ultrasound

Pulsed-wave Doppler measurements were performed at the CFA, SFA, and DFA using a conventional clinical US system with a linear 14L5 transducer (ACUSON S2000; Siemens AG, München, Germany). Clinical guidelines for vascular laboratory examinations^30^ were followed: velocities were obtained at the center of the lumen with a sampling volume of 1 mm and a pulse repetition frequency of 13 kHz. The beam-to-velocity angle was kept below 60 deg.

### 2.6 Computational Fluid Dynamics

The SimVascular finite-element solver (Stanford & UC Berkeley, Palo Alto & San Francisco, California) was used for CFD simulation of the two geometries. This open-source solver has been optimized for cardiovascular flows with pre-conditioners accounting for resistive or Windkessel boundary conditions and stabilization of reversed flow in the outlets.^31^ The geometries were segmented from the CT-scans of the PDMS models, using a regularized level-set algorithm.^32^ Tetrahedral elements were used to construct a volumetric mesh with a three-element prism boundary layer to capture the high shear gradients near the wall. The vessel wall was considered rigid and the density and dynamic viscosity of BMF were used as material properties.

For both geometries, a mesh convergence study was performed for peak systolic flow rate. Convergence was defined as an overall smaller than 3%-change for the centerline velocities in the CFA and SFA for a doubling of the mesh points. Mesh convergence for the stenosed geometry, in which transitional flow was present (characterized by significant turbulent kinetic energy), was achieved for the time-averaged velocity along the centerline (Appendix C).

For the two geometries, the flow boundary conditions were derived from both DUS and ePIV, as these techniques are suitable for obtaining flow boundary conditions in patients. For DUS, the centerline velocity was converted to a flow rate over time, by using Womersley theory under the assumption of a fully developed flow profile and the vessel diameters measured on CT.^33^ For ePIV, which provides a 2D spatial velocity profile along the diameter in the CFA, SFA, and DFA, the flow rate was computed as follows:

$$Q(t)=\pi \int_{-R}^{R}u(r,t)|r|dr,$$ (1)

where Q is the flow rate, R is the radius of the cross-section, and u is the axial velocity. With the velocity only known for polar angle θ=0 and θ=π, this equation assumes that the angle-averaged velocity for a radial coordinate is equal to the average of the values measured at θ=0 and θ=π.

### 2.7 Analysis of oPIV, ePIV, and CFD Data

For all modalities, 10 cardiac cycles were aligned at peak systole and ensemble-averaged over time to compare the characteristics of the mean transitional flow. The cycle-averaged data were used to extract various flow parameters including spatial and temporal velocity profiles, VC (see below), and WSS. The spatial and temporal velocity profiles were obtained at the CFA, SFA, and DFA of the control model, and at mid stenosis and at the post-stenotic jet of the stenosed model. For all temporal velocity profiles, the absolute peak differences between the three modalities relative to oPIV were calculated.

To statistically assess the agreement of the three techniques relative to oPIV, velocity vector data from ePIV and CFD were resampled using linear interpolation (i) in space at the vector locations of the oPIV grid and (ii) in time to 100 sample points of the averaged cardiac cycle. The mean and standard deviation (SD) of the difference in velocity magnitudes for all spatiotemporal velocity vectors, referenced to oPIV values, were calculated to assess the agreement in velocity in space and time between the modalities. This was repeated for all velocity vectors during peak systole to assess the agreement in space between the modalities at this single timepoint. The limits of agreement were defined as mean ±1.96 SD, covering 95% of the area under a normal curve.^34^

Two flow-related parameters were derived from the velocity vector fields and compared between the modalities. First, VC was obtained as a metric to describe the distribution in vector direction in which completely unidirectional parabolic flow will have a value of 0 and recirculating complex flow with a large spread in vector direction will have a value toward 1.^35^^,^^36^ VC was computed as follows:

$$\mathrm{VC}=1-\sqrt{{\overset{¯}{x}}^{2}+{\overset{¯}{y}}^{2}},$$ (2)

and

$$\overset{¯}{x}=\frac{1}{n}\overset{n}{\underset{i=1}{\sum }}\;\cos\;\theta_{i},$$ (3)

$$\overset{¯}{y}=\frac{1}{n}\overset{n}{\underset{i=1}{\sum }}\;\sin\;\theta_{i},$$ (4)

$$\theta_{i}=\tan^{-1}(v_{x,i},v_{y,i}),$$ (5)

where $\theta_{i}$ is the beam-to-flow angle of the vector $v_{x,i},v_{y,i}$ at position i, $\overset{¯}{x}$ and $\overset{¯}{y}$ are the averaged beam-to-flow angles represented on the unit circle, and n is the total number of vectors in the region of interest. VC was derived in a region around the stenosis, i.e., 1 cm upstream until 1 cm downstream of the stenosis. The same area was selected in the control model. Second, time-averaged WSS (TAWSS) was determined for the inner and outer walls of the CFA and SFA for the oPIV and ePIV data by interpolating the wall-parallel velocity component along a line perpendicular to the wall. Subsequently, a weighted cubic spline was fitted to the velocity component parallel to the wall and used to calculate TAWSS.^37^ For both CFD simulations, TAWSS was provided by the solver based on the shear rate tensor.

## 3 Results

### 3.1 Control Model

**Fig. 2:**
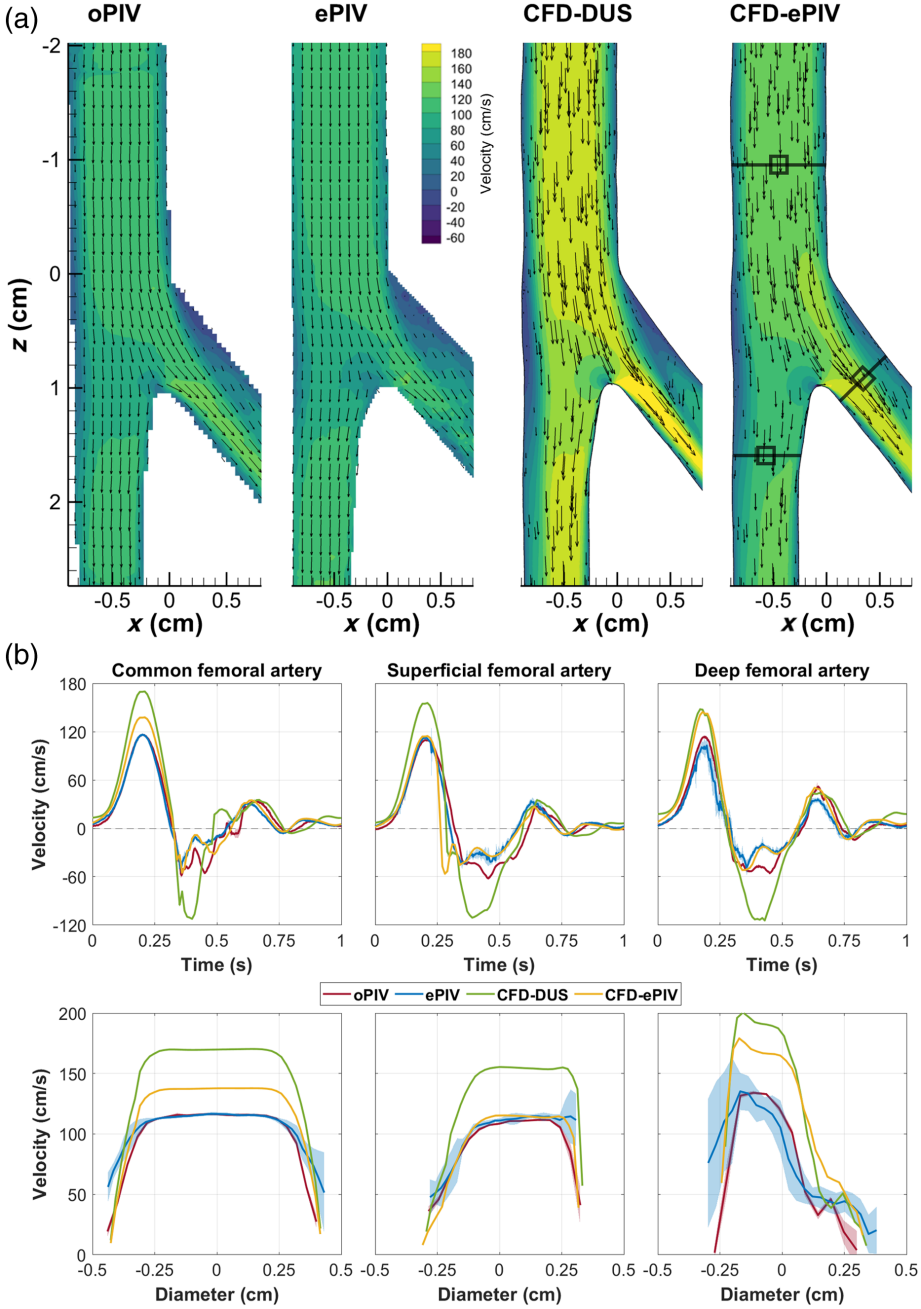
(a) In-plane velocity vector fields at peak systole in the control model. Black line and square indicate the locations where (b) velocity profiles were extracted. Cycle-averaged temporal (upper row) and spatial (lower row) velocity profiles extracted at (from left to right) the CFA, SFA, and DFA. Shading represents the standard deviation over 10 cardiac cycles. Video 1 shows animations of (a) over time and Appendix D provides spatially averaged velocity profiles of (b).

Qualitatively, similar flow features were observed in the control model for ePIV, CFD-DUS, and CFD-ePIV compared to the oPIV reference [Fig. 2(a) and Video 1]. For all approaches, a flow separation at the outer SFA and DFA walls near the bifurcation was observed during forward flow with high velocities at the inner walls of the SFA and DFA. During backflow, the oblique flow originating from the DFA interacted with the SFA flow and created undulating flow structures. These flow structures were slightly less defined for ePIV, yet exaggerated and with a phase-offset for CFD-DUS.

Temporal and spatial velocity profiles of the CFA showed moderate to good agreement with an absolute peak velocity difference of 0.19  cm/s between oPIV and ePIV and 21.6  cm/s between oPIV and CFD-ePIV [Figs. 2(b) and Appendix D]. CFD-DUS showed higher velocities with an absolute peak difference of 53.7  cm/s compared to oPIV and a larger diastolic phase off-set. Near the wall, both CFD approaches followed the oPIV curve with a sharp transition toward lower velocities of ∼10  cm/s, whereas the ePIV curve appeared smoother and velocities remained relatively high around 50  cm/s. For the SFA, absolute peak velocity differences were 2.9, 46.0, and 5.0  cm/s for ePIV, CFD-DUS, and CFD-ePIV, respectively, with respect to oPIV. The corresponding spatial velocity profiles presented higher velocities at the inner wall of the SFA due to the bifurcation. For the DFA, larger deviations were found between oPIV and ePIV, and between oPIV and CFD-ePIV, with absolute peak differences of 11.0 and 30.7  cm/s, respectively. The difference between oPIV and CFD-DUS was slightly smaller compared to the other locations with a peak difference of 34.0  cm/s. All modalities showed a similar trend in the spatial velocity profiles with a steep gradient at the inner wall of the DFA. Mean differences of the entire CFA-SFA region averaged over all time points, referenced to oPIV, are minimal for ePIV and CFD-ePIV with similar limits of agreement (Table 1). The spatial plot of the SD [Fig. 4(a)] demonstrates the highest spread for ePIV and CFD-ePIV relative to oPIV in areas with high velocities during backflow. CFD-DUS showed an overall larger disagreement with oPIV.

**Table 1:** Limits of agreement (mean ±1.96 SD) for all vector points in the CFA and SFA between the three modalities relative to oPIV.

		All timepoints (cm/s)	Peak systole (cm/s)
Control	ePIV	−2.9 ± 15.0	0.8 ± 18.9
	CFD-DUS	13.0 ± 40.7	34.0 ± 27.6
	CFD-ePIV	−0.3 ± 24.8	−0.3 ± 27.2
	ePIV	1.5 ± 33.5	10.7 ± 57.1
Stenosis	CFD-DUS	16.1 ± 18.9	48.3 ± 102.7
	CFD-ePIV	−0.1 ± 33.8	−0.1 ± 59.1

The mean magnitude for all velocity vectors in the oPIV model was 25.6 and 35.6  cm/s for the control and stenosed model, respectively.

### 3.2 Stenosed Model

**Fig. 3:**
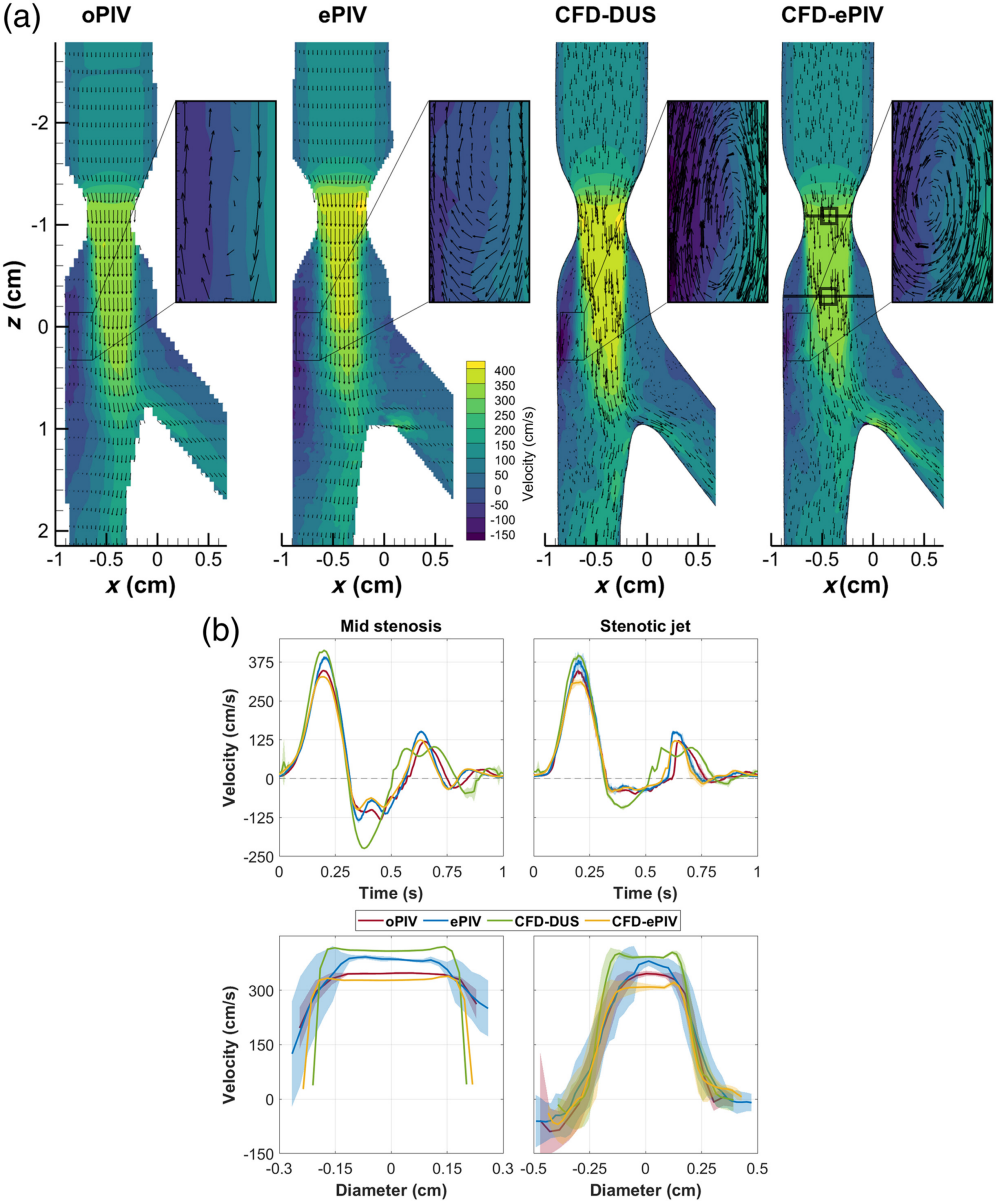
(a) In-plane velocity vector fields at peak systole in the stenosed model. (b) Similar spatiotemporal overview as Fig. 2, but for a location in the stenosis and downstream in the post-stenotic jet, as indicated by the black markers. Video 2 shows an animation of (a) over time and Appendix D provides spatially averaged velocity profiles of (b).

**Fig. 4:**
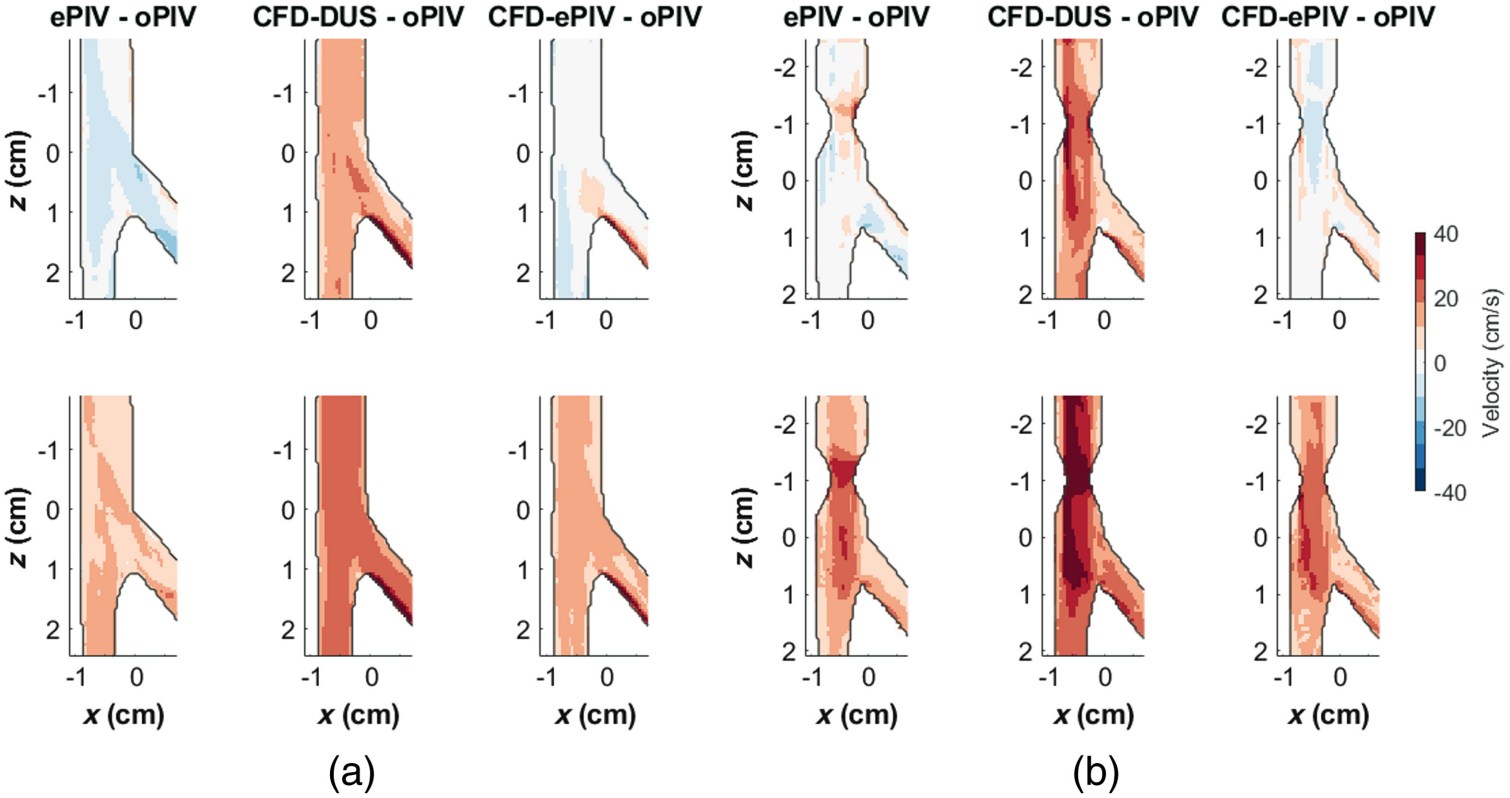
Mean differences (top) and standard deviation (bottom) in velocity magnitudes over the entire cardiac cycle between ePIV, CFD-DUS, and CFD-ePIV relative to oPIV in (a) the control model and (b) the stenosed model.

Similar flow patterns were observed in all techniques in the stenosed model [Fig. 3(a) and Video 2]. All approaches showed the post-stenotic jet extending up to the bifurcation point during forward flow. High spatial velocity gradients were captured at the jet border, especially at the left side where strong reversed flow velocities were present [inset Fig. 3(a)]. Post-stenotic recirculation zones were captured by all modalities. During flow reversal, all techniques showed similar dynamics including a reversed jet into the CFA with associated recirculation zones. Higher stenotic velocities, with an absolute peak difference of 57.7 and 57.0  cm/s, respectively, were captured by ePIV and CFD-DUS compared to oPIV [Fig. 3(b) and Appendix D]. CFD-ePIV showed better agreement with oPIV, with an absolute peak difference of 23.2  cm/s. During diastole, CFD-DUS presented a phase off-set, whereas the other approaches were well-aligned. No large cycle-to-cycle variations were found at the centerline velocity of the jet, whereas closer to the wall, at locations with strong spatial gradients, this variation increased. The spatial profile captured by ePIV was more parabolic compared to the flattened profile of the other techniques but estimated similar low near-wall velocities at the level of the jet. For the stenosis, however, ePIV measured increased near-wall velocities with a larger cycle-to-cycle variation. Overall, mean differences showed minimal bias in ePIV and CFD-ePIV velocity magnitudes, with a moderate positive bias for CFD-DUS [Figs. 4(b) and Table 1]. For ePIV, the largest differences and spread were found specifically at the inlet of the stenosis, whereas the entire stenosed region showed small positive biases for CFD-ePIV. The largest differences for CFD-DUS were found in a similar region inside and around the stenosis. Here, the variation over time was elevated as well with increased limits of agreement compared to the control model and compared to the other modalities.

### 3.3 Vector-Derived Parameters

**Fig. 5:**
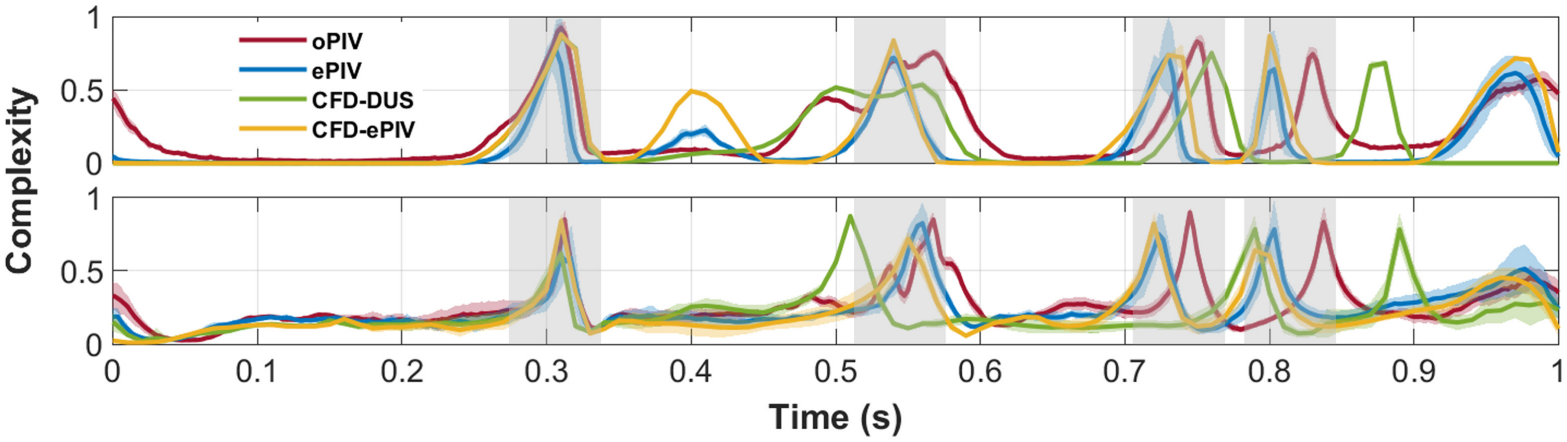
VC over time extracted in the CFA of the control (top) and stenosed (bottom) model. Periods of flow reversal are indicated in grey. Shading represents the standard deviation over ten cardiac cycles.

VC showed good agreement between all techniques for both models, except for the diastolic phase offset and corresponding time mismatch in peaks during flow reversal (Fig. 5). In the control model, mean peak systolic VC was <0.01 for all techniques. In the stenosed model, the mean peak systolic VC was elevated compared to the control model and equaled 0.17±0.04, 0.16±0.02, 0.13±0.04, and 0.12±0.02 for oPIV, ePIV, CFD-DUS, and CFD-ePIV, respectively. When only the post-stenotic recirculation was analyzed, excluding the jet, VC increased to 0.69±0.20, 0.75±0.15, 0.34±0.13, and 0.41±0.15.

**Fig. 6:**
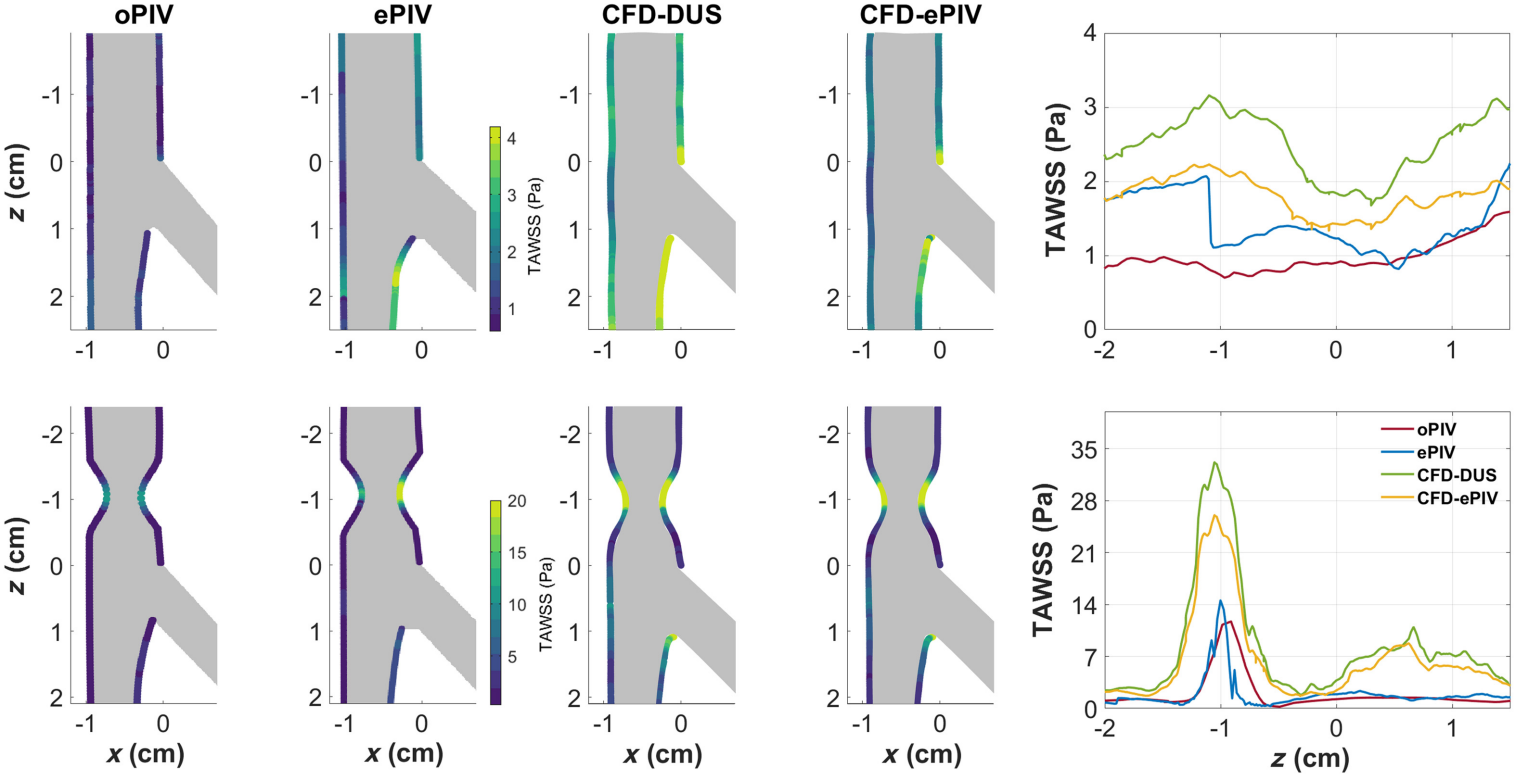
In-plane TAWSS at the CFA and SFA walls for the control (top) and stenosed (bottom) model obtained with all modalities. A comparison of the CFA TAWSS between z=−2 and z=1.5  cm between all modalities is given on the right-hand side.

Overall higher TAWSS values were found with ePIV, CFD-DUS, and CFD-ePIV compared to oPIV in the control model (Fig. 6). Lowest WSS was located at the CFA wall where the DFA splits off, whereas the highest WSS was found at the inner SFA wall. Mean TAWSS for the segment given in Fig. 5(e) was 1.43, 2.50, and 1.80 Pa for ePIV, CFD-DUS, and CFD-ePIV, respectively, compared to 0.97 Pa for oPIV. For the stenosed model, highest TAWSS values were found within the stenosis for all techniques. At this location (around z=−1  cm), both CFD approaches measured two- to threefold higher TAWSS values compared to oPIV. For ePIV, similar values to oPIV were found for the left CFA vessel wall, yet a twofold increase in TAWSS was present at the right CFA vessel wall. This asymmetry was not seen in the other techniques. On average, the TAWSS of the left CFA-SFA vessel wall was 1.68, 8.68, 6.76 Pa for ePIV, CFD-ePIV, and CFD-DUS, respectively, compared to 1.68 Pa measured with oPIV.

## 4 Discussion

The accuracy of blood flow assessment was investigated in models of the femoral bifurcation by clinically applicable ePIV measurements and CFD simulations. Using oPIV measurements as the gold standard, ePIV and CFD-ePIV demonstrated good overall agreement in vector velocities in a stenotic flow with limits of agreement of 0.1 to 1.5±34  cm/s. The more clinically available CFD-DUS approach was less accurate with limits of agreement of 16±55  cm/s in the stenosed model. For the vector-derived parameters, good agreement was found for VC between all four techniques in regions with laminar flows as well as regions with complex flow phenomena, such as a recirculation. TAWSS showed reasonable agreement for all modalities in the control model. Larger deviations of TAWSS were found in the stenosed area, whereas good agreement was found outside of this region for ePIV. The accuracy of the oPIV-reference for TAWSS assessment was debatable, however.

All three approaches demonstrated largely similar flow dynamics in the control and stenosed model. The complex transitional dynamics of the post-stenotic jet were well captured by the ePIV and CFD techniques, including its breakdown, recirculating flows near the jet and high spatial velocity gradients at the border of the jet. This generates confidence for using these techniques to assess complex flow velocities in clinical practice in general and more specifically in patients with PAD.

Several disagreements with oPIV were present. For ePIV, spatial velocity profiles in the post-stenotic jet and in near-wall regions had a smaller gradient compared to oPIV. In addition, velocities measured within the stenosis were higher than obtained with oPIV. The differences in the spatial velocity profiles can be attributed to smoothing by intrinsic factors that include spatial averaging by the UCA point-spread function, which is larger than the speckle size, and by the PIV correlation kernel dimensions.^38^ The latter could be improved by using anisotropic kernels aligned with the spatial velocity gradients. The Gaussian spatial averaging in the post-processing step likely also contributed to these differences, but this step is required to suppress noise and is therefore part of the intrinsic limitation of the technique, so far.^16^ The increased velocities within the stenosis for ePIV possibly originated from a difference in compliance between the optical and acoustical models. The thin-walled ePIV resin model exhibited less pulsatile wall motion (0.5% to 1% as observed by pixel displacement) than the oPIV PDMS block model (2% to 4% as observed by pixel displacement), which led to a larger stenotic diameter in the oPIV phantom during peak systole relative to the ePIV phantom. Under the same peak flow rate, this would be associated with higher velocities in the ePIV phantom. The difference in phantom compliance furthermore led to a subtle offset in timing of the backflow and subsequent forward flow, which could explain the distribution of velocity discrepancies in the bifurcation region.

For CFD-ePIV, steep gradients in velocity profiles were sufficiently sampled, but still similar limits of agreement as ePIV were found in the order of ±25  cm/s (control) and ±34  cm/s (stenosed) relative to oPIV. Judging from the evolution of the temporal velocity profiles [e.g., Figs. 2(b), 2(c) and Figs. 3(b), 2(c)], an important contributor to this spread was the slight offset in timing of the backflow and subsequent forward diastolic flow. Here, the difference in compliant behavior was likely responsible too. Second, peak forward flow was overestimated in the control model by CFD-ePIV, possibly due to a positive bias of the flow rate estimation by ePIV, as higher near-wall velocities were registered by ePIV. This has an important contribution to the overall flow rate estimation as velocities are multiplied by the radius (Eq. 1). For CFD-DUS, an even larger positive bias in the velocity magnitude was present, as well as important differences in the phase and velocity profile of backflow and diastolic flow. These differences can be explained by spectral broadening^39^ and finite transit time.^6^ Clinical DUS examinations that we followed, especially for the small sampling volume of 1 mm, have been associated with peak velocity overestimation of 10% to 50%, depending on the device used.^6^ The variability in errors between devices, as well as the high interobserver variability of 20% for the SFA,^8^^,^^9^ makes it hard to systematically correct for these errors in clinical scenarios. The phase difference in backflow resulted from deviations from a (theoretical) Womersley profile during this phase, due to reversed flow through the bifurcation creating strongly asymmetric spatial flow profiles.

VC was very high during flow reversal phases, which complicates interpretation of this parameter for vessels that exhibit backflow. Instead, a focus on peak systole could be relevant to quantify areas with disturbed flow, as VC was consistently higher at this time point for the stenosed area relative to the control model. TAWSS demonstrated similar profiles along the CFA and SFA wall, with an overall increase in magnitude for all three modalities compared to oPIV in the control model and inside the stenosis. This positive bias in TAWSS for ePIV might in part be caused by the higher near-wall velocities in ePIV, as discussed previously. Another potential factor is the finer vector grid spacing of ePIV, which could also explain the asymmetry in TAWSS values for the stenotic model in ePIV. A smaller distance between the nearest wall velocity for the right wall relative to the left wall could have led to increased TAWSS at the right wall. The CFD approaches showed overall increased TAWSS values. For the PIV techniques, a suboptimal resolution for the near-wall velocities^40^ and the chosen curve fitting method are known to impact TAWSS estimates.^41^ These factors limited oPIV assessment of TAWSS, making it hard to judge to what extent ePIV and CFD approaches were inaccurate and to what extent the oPIV reference was truthful. This could be improved by using smaller camera windows and using interfacial PIV, which provides more accurate, higher TAWSS values relative to the standard oPIV applied in this study.^19^

While ePIV was able to measure TAWSS in the physiological order of magnitude, translation into the clinic remains challenging as a precise detection of the vessel wall and corresponding segmentation of the flow lumen is difficult. Furthermore, current vector resolution limits the accurate assessment of near-wall velocities. For CFD approaches, the clinical application for estimating TAWSS can be more straightforward.

This study extends previous experiments that have validated ePIV and finite-element CFD in various geometries and modeling strategies. These include ventricular,^14^ aortic,^42^^,^^43^ coronary,^23^ and carotid^15^^,^^22^ artery phantoms. For ePIV, the differences in velocity magnitude of about 9% to 17% have been reported,^14^^,^^15^ which was of similar order in our study (4% to 11%). For CFD, we used flow boundary conditions from ePIV or DUS, instead of the benchmark technique (oPIV or PC-MRI). Here, we wanted to study the technical validity of the CFD methodology, where our approach relies on ultrasound-based methods that can be applied with low cost (DUS) or with more advanced ultrasound imaging (ePIV). Furthermore, our study supports the validity of an under-resolved direct numerical simulation strategy for estimating cycle-averaged stenotic flows, as previously reported for a carotid artery stenosis.^22^

Future validation studies can focus on a comparison of the ePIV and CFD techniques to other clinically available vector flow imaging modalities. These include PC-MRI, ultrasound blood speckle tracking techniques, vector Doppler techniques, and 3D-ultrasound.^13^^,^^44^ Depending on patient anatomy, flow conditions, and clinical purpose, all techniques have different strengths and weaknesses, which will require different and possibly synergistic approaches where necessary. PC-MRI can provide time-resolved 3D velocity fields and is less susceptible to calcifications but is relatively limited in spatial and temporal resolution, prone to stent artifacts, and requires interleaved sampling, which filters details on cycle-to-cycle variations of complex flow behavior and may be challenged by variable heart rates.^45^ Native blood speckle tracking techniques are cost-effective, but may be less attractive due to limited image contrast for deeper-lying vessels.^46^ CFD approaches can provide high-resolution 3D velocity fields and WSS at low additional cost, but its accuracy will depend on input and output flow boundary data and the accuracy of CFD computed WSS is uncertain.

The *in vitro* validation of vector flow imaging modalities is an important step toward its potential implementation in clinical practice. The 2D or 3D visualization and quantification of blood flow characteristics could provide in-depth insights in local hemodynamics superior to DUS examination as used in current clinical decision making. In addition, vector derived parameters, such as VC and WSS, could be used to quantify complex flow phenomena and identify locations potentially at risk for plaque formation, in-stent restenosis^47^ or stentgraft thrombosis.^48^ An extra potential benefit of CFD models is the ability to predict the effect of different treatment strategies on local hemodynamics to select a treatment that creates an optimal hemodynamic environment, with the aim to reduce reintervention rates.^49^^,^^50^ The feasibility of ePIV and CFD in patients with atherosclerosis has already been shown in small patient populations.^16^^,^^47^^,^^48^^,^^51^^,^^52^ However, longitudinal studies with larger cohorts are necessary to show the potential benefit of these novel modalities in clinical practice and to study the relation between vector derived parameters and clinical outcome.

This study has several limitations. The most important limitation was the use of different models for the oPIV and ePIV measurements due to incompatible transmission constraints. Care was taken to minimize differences in anatomic parameters (±0.25  mm) and flow rate (±0.1  mL/s), and furthermore a difference in compliant behavior (2% to 3% more radial distension) was present. It can be estimated that in the CFA, a 5.6% difference in radius would lead to an 11.5% decrease in velocity in the oPIV phantom relative to ePIV, with an additional 6% decrease during systole due to the increased pulsatility of the oPIV model. In the stenosed CFA, the same radial difference could cause a 23% difference in velocity and could explain the elevated velocities in the ePIV model, with similar implications for the TAWSS. Furthermore, although the US and laser plane were carefully positioned, slight misalignment could have been present and have influenced the outcomes. Also, the PIV analysis differed slightly between both modalities. Equal kernel sizes were used for oPIV and ePIV, yet ePIV had a finer grid because of the 75% overlap (oPIV 50% overlap). A 75% overlap was not available in the PIVlab toolbox that was used for the oPIV analysis. Differences in grid spacing were offset in the statistical assessment between the modalities, as all grids were interpolated on the oPIV grid. Furthermore, the effects of non-arterial tissue and imaging artefacts by calcifications, stents or other pathologies were not investigated, which can constitute major limitations for ePIV imaging in patients^16^ or for CTA segmentation^53^ as input for the CFD approaches. Last, although all ultrasound hardware, pulse characteristics, and post-processing steps were set according to clinical use, tissue attenuation was not taken into account.

## 5 Conclusion

This study has shown that ePIV and CFD-ePIV can accurately estimate complex stenotic transitional flows. CFD-DUS mostly overestimated the velocity magnitudes, which can be attributed to DUS measurement inaccuracies and the limited information that it provides for a flow rate reconstruction. Nevertheless, similar post-stenotic and bifurcation related flow patterns were observed between all modalities with comparable VC values and trends in TAWSS. Although the applied ePIV and CFD techniques require further development for a routine application in clinical care, they are attractive from a cost-effectiveness perspective and provide the ability to assess hemodynamic insights with high temporal and spatial resolution.

## 6 Appendix A: Averaged Anatomy and Flow Curve

### 6.1 Anatomy

The diameters of the three femoral arteries were based on a literature study, yielding the characteristics as shown in Table 2. The weighted mean and weighted standard deviation of the reported cohorts were computed. The mean diameters of the three arteries were used to design the femoral bifurcation phantom models. For the SFA, a diameter of 6.2 mm instead of 6.3 mm was used.

**Table 2:** Literature review on femoral artery diameters.

	Diameter (mm)	Cohort size	Sex	Modality	Reference
CFA	8.9 ± 1.5	122	63 female	Ultrasound	54
	*8.9 ± 1.5*	*122*			
SFA	5.7 ± 1.2	55	25 female	Ultrasound	55
	6.9 ± 1.3	18	9 female	Ultrasound	56
	6.2 ± 0.9	59	Not reported	IVUS	57
	7.7 ± 1.5	22	10 female	Caliper	58
	*6.3 ± 1.5*	*154*			
DFA	6.0 ± 1.0^a^	228	Not reported	Caliper	59
	6.9 ± 1.6	22	10 female	Caliper	58
	*6.1 ± 1.1*	*250*			

a Based on reported normal range. IVUS, intravascular ultrasound.

Note: Values in Italics show the weighted mean and weighted standard deviation.

### 6.2 Flow Curve

Pulsed-wave Doppler ultrasound (DUS) measurements were performed at the CFA, SFA, and DFA in seven healthy participants using a conventional clinical US system with a linear 9L3 transducer (iU22; Philips, Amsterdam, The Netherlands). Clinical guidelines for vascular laboratory examinations were followed: velocities were obtained at the center of the lumen with a sampling volume of 1.5 mm and a pulse repetition frequency of 2.1 to 4.2 kHz. The beam-to-velocity angle was kept below 60 deg.

The measurements of the DFA were of mediocre quality (broad spectrum with unclear peak velocity), so only the measurements of the CFA and SFA were used. Under the assumption of fully developed Womersley flow, the flow rate was computed similar as described for the DUS measurements in the paper methods using Eq. (1). Because of significant differences in interval between peak forward and backward flow, a median value of this interval in the seven participants was computed. The timescale of the flow rate curves was rescaled so that all had an interval equal to this median value. Subsequently, the ensemble average curve was computed.

**Fig. 7:**
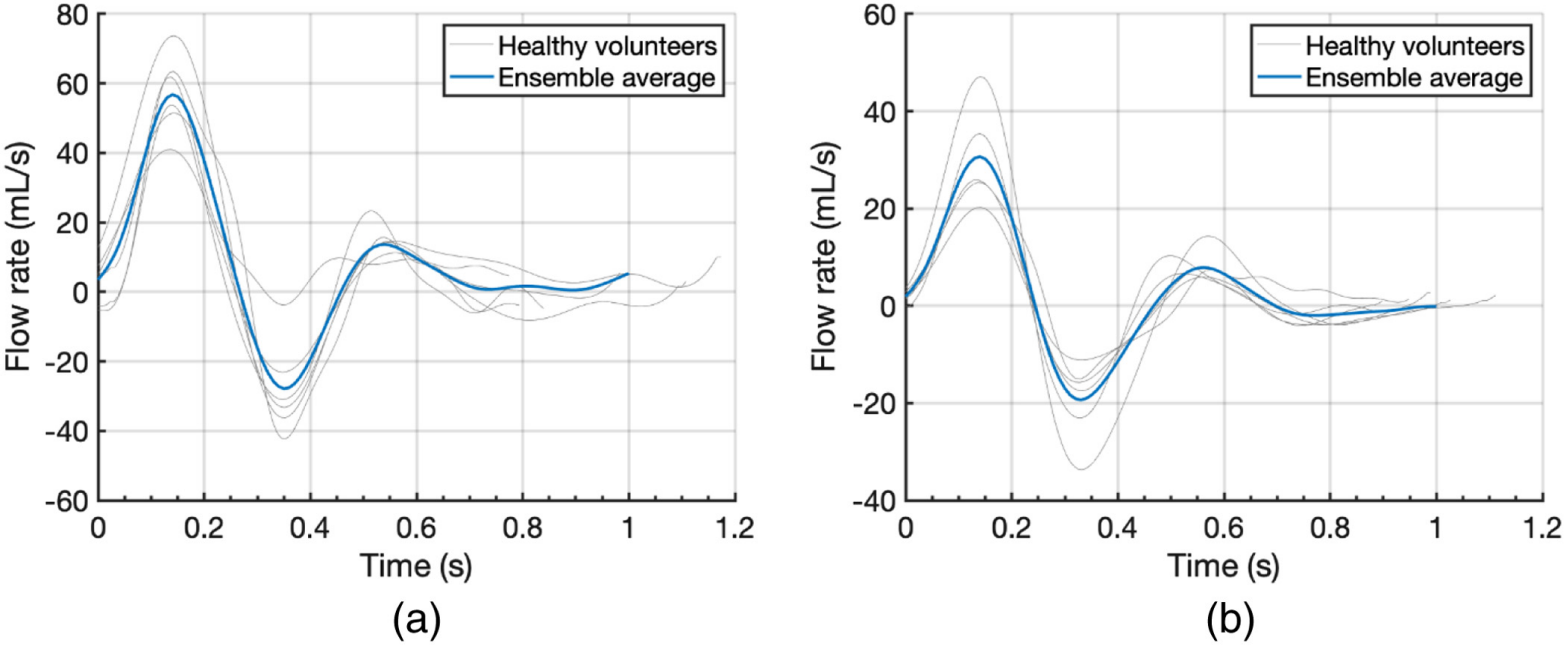
Flow rate of the (a) CFA and (b) SFA.

The CFA curve served as reference for the inflow boundary condition (Fig. 7). The DFA flow rate was considered equal to the CFA minus SFA flow curve. Together with the SFA curve (Fig. 7), they were used to compute the mean flow split and the capacitance split for the outflow Windkessels.

## 7 Appendix B: Phantom Geometries

**Fig. 8:**
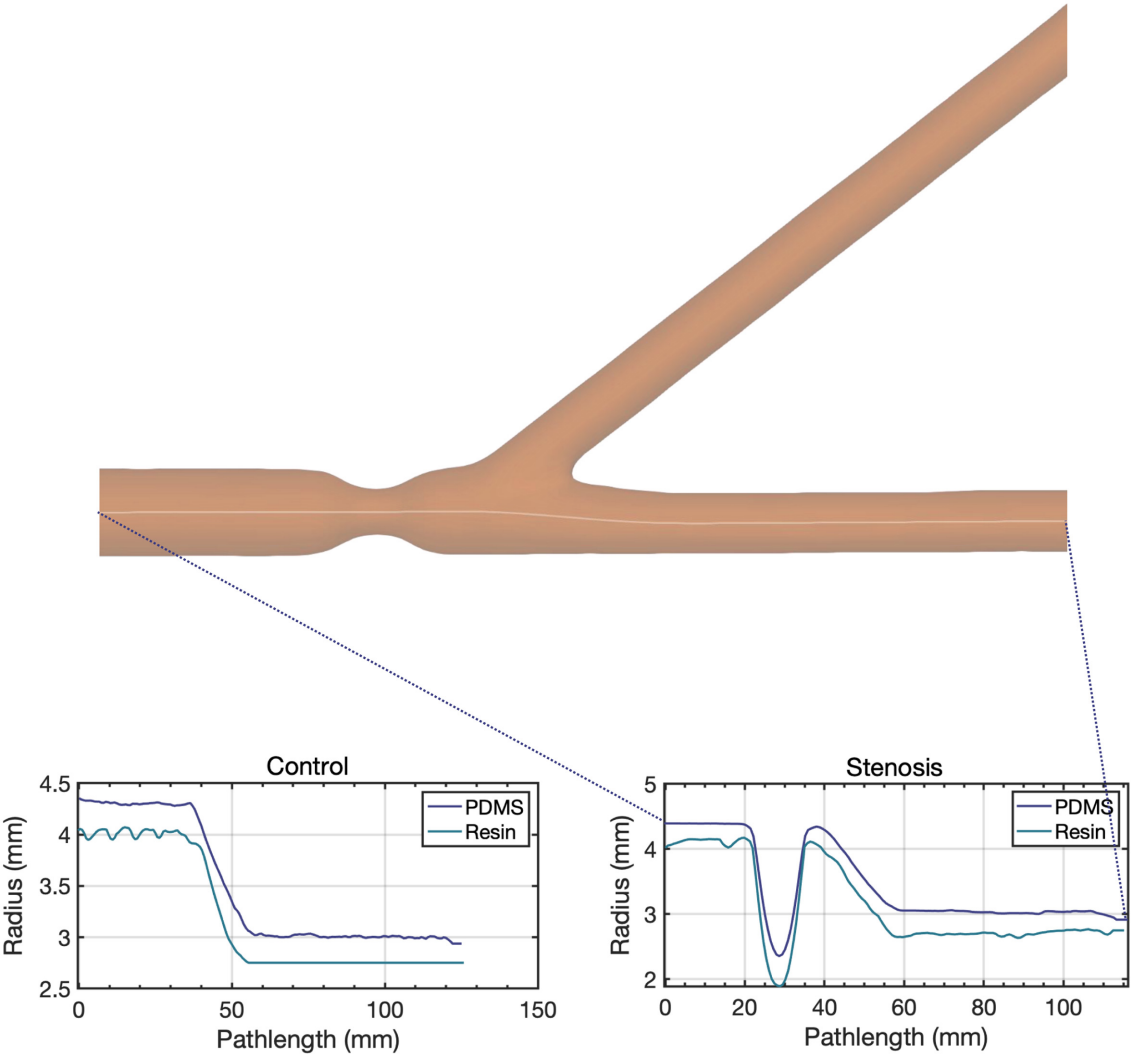
Segmented stenosed geometry with the computed centerline for the CFA and SFA. The vessel radius of the two models (oPIV; PDMS and ePIV; resin) along this centerline for control and stenosed model are shown in the plots.

From the cone-beam CT scan, the lumen of the oPIV en ePIV models was segmented with the VMTK level-set algorithm^32^ based on the contrast between the wall and the lumen filled with air. With the inscribed-sphere VMTK algorithm, the radius along the centerline of the models was assessed. The radius along this centerline for the models is shown in Fig. 8.

## 8 Appendix C: CFD Mesh Convergence for Stenosed Bifurcation

For the stenosed model, the convergence of a steady inflow, equal to the peak systolic flow rate (54.2  mL/s), was assessed for three meshes. The mesh convergence for velocity, pressure, VC, and wall shear stress was assessed. Mesh size was set such that mesh 2 included roughly twice as much points as mesh 1, and vice versa for mesh 3 relative to mesh 2. For all meshes, a regional mesh refinement (twice as many points per volume) was applied within a spherical volume that included the stenosis and its downstream region extending up to the bifurcation. The approximate mesh characteristics were as follows.

Mesh 1: 1.54 million cells, 270 thousand points, 0.46 mm edge length in stenosis region;

Mesh 2: 2.64 million cells, 453 thousand points, 0.36 mm edge length in stenosis region;

Mesh 3: 4.98 million cells, 843 thousand points, 0.29 mm edge length in stenosis region.

For the applied steady inflow rate, velocities in the stenotic jet of about 310  cm/s were expected. This is associated with a Reynolds number of 3780 in the region downstream of the stenosis:

Re=vρLμ=31002·1.14·10−6·8.94.16·10−6,

where v is the spatial mean velocity, ρ is the fluid density, L is the local diameter, and μ the viscosity, all in mm, kg, and s. Under Kolmogorov homogeneous isotropic turbulence theory, the associated length scale of the smallest flow structures would be

η=ν3ϵ14=lcRe34=0.019  mm.

Thus, for mesh 3, the edge length in the area of transitional flow is one order of magnitude higher than the estimated Kolmogorov length scale under isotropic turbulence.

**Fig. 9:**
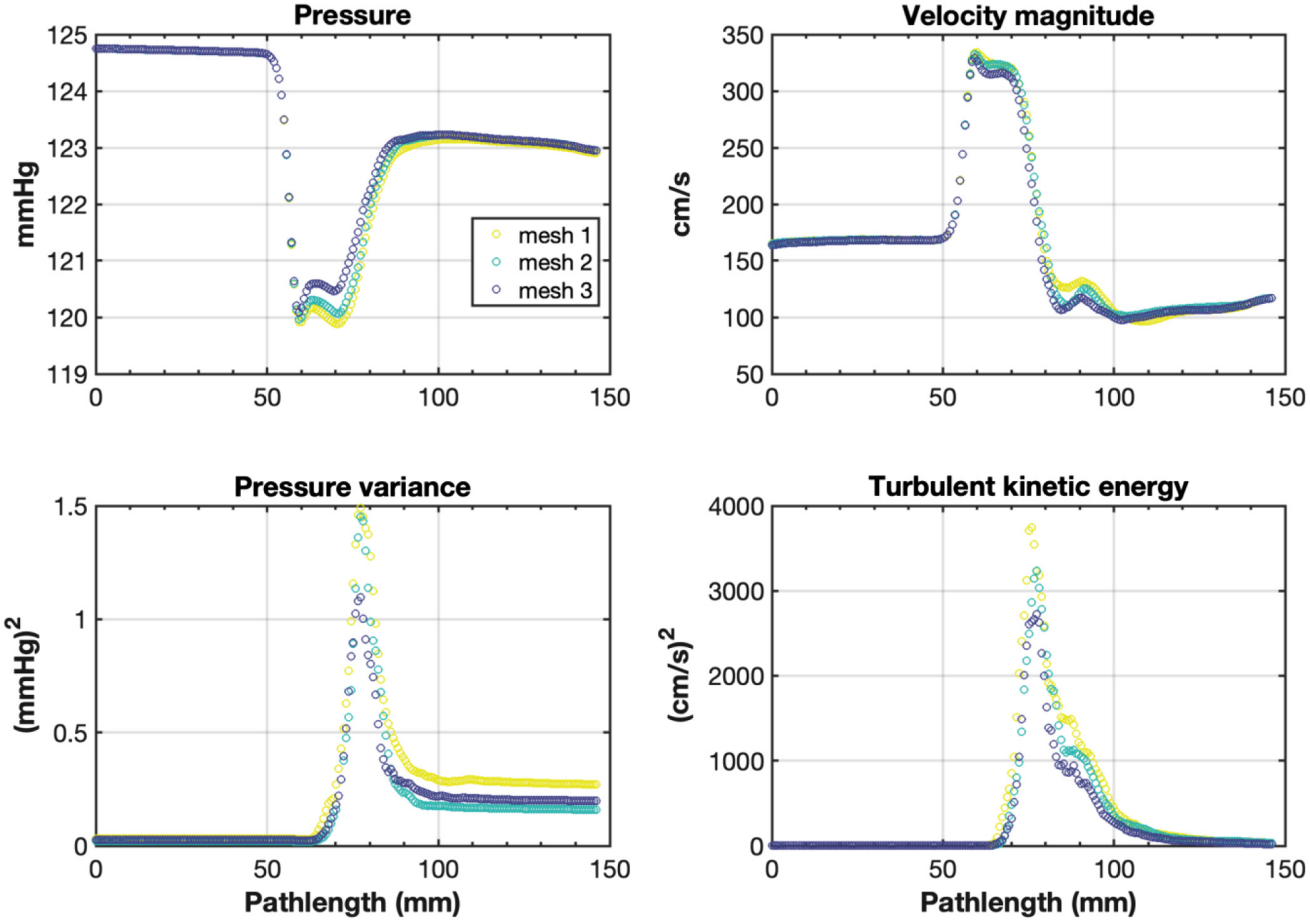
Mesh convergence study for points along the CFA and SFA centerline in the stenosed model.

For the points along the common femoral–SFA centerline (see Appendix B), the temporal transitional flow statistics were assessed by computing the temporally averaged solution and the variance for the pressure and velocity magnitude variables (Fig. 9). For the mean velocity and pressure field, mesh 2 was considered converged relative to mesh 3 (mean absolute difference along centerline in pressure of 0.2% and in velocity of 2.2%, with a maximum point velocity difference in post-stenotic area of 8.5%). Nonetheless mesh 3 was used for further (pulsatile) simulations.

From the pressure variance and turbulent kinetic energy (Fig. 9), it can be observed that the mesh was not converged for the fluctuating components of the turbulence, with the turbulent kinetic energy decreasing for more refined meshes, where a mean spatial absolute difference along the centerline in turbulent kinetic energy of 21.2% was present between mesh 2 and mesh 3.

For VC, the same region of interest as in the main method that included the stenosis and the post-stenotic jet was used for analysis. For the three meshes, the value of VC was 0.121, 0.117, and 0.117 for mesh 1, mesh 2, and mesh 3, respectively.

For wall shear stress, the left wall that spanned the CFA, the stenosis, and the superficial artery was used for analysis. The average wall shear stress along this wall was 8.68, 9.20, and 9.87 Pa for mesh 1, mesh 2, and mesh 3, respectively.

## 9 Appendix D: Temporal and Spatial Velocity Profiles

**Fig. 10:**
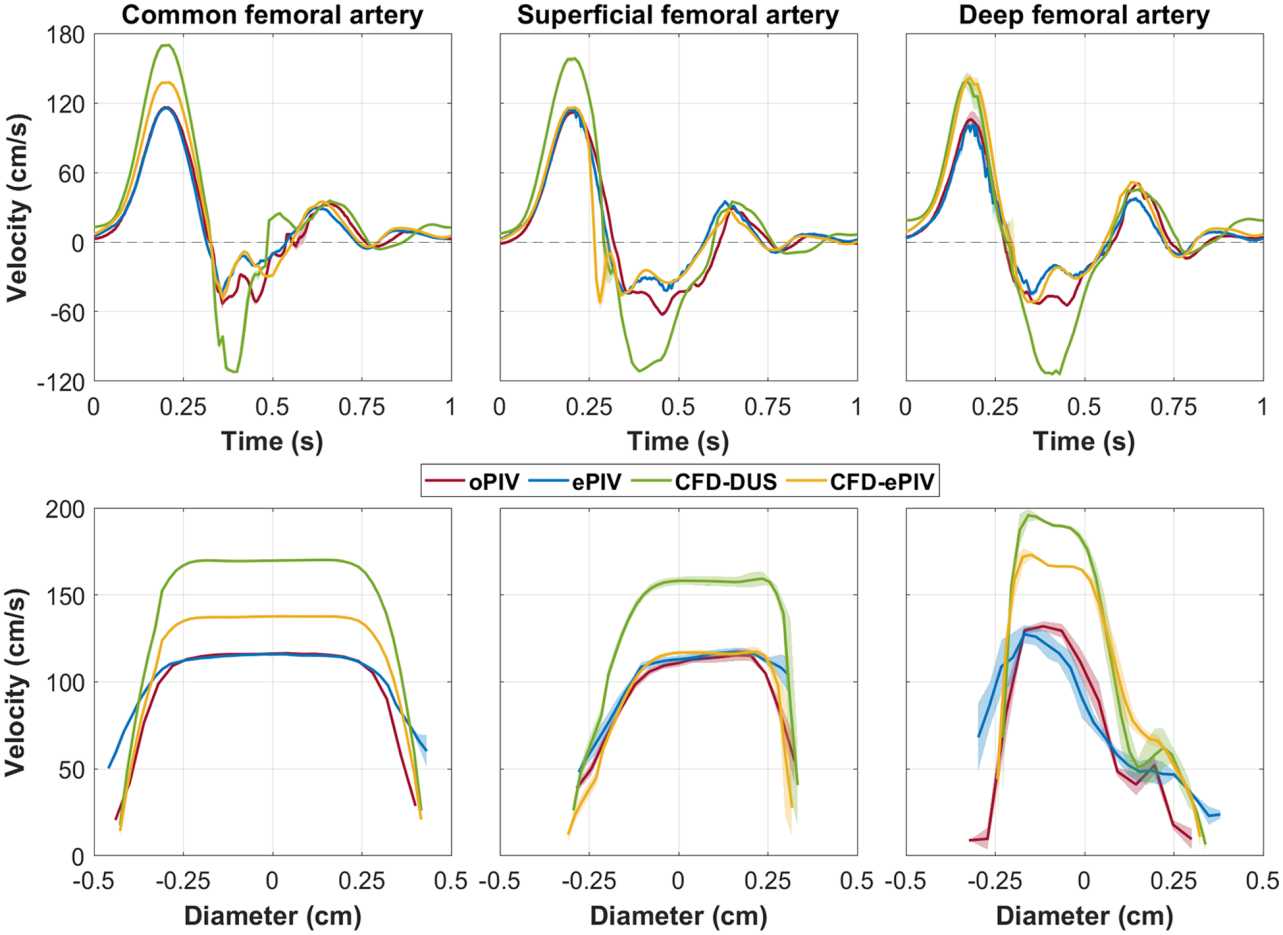
Temporal (left) and spatial (right) velocity profiles of the CFA, SFA, and DFA in the control model. Shading represents the standard deviation in space probed over five points at a 0.5 mm distance of each other.

**Fig. 11:**
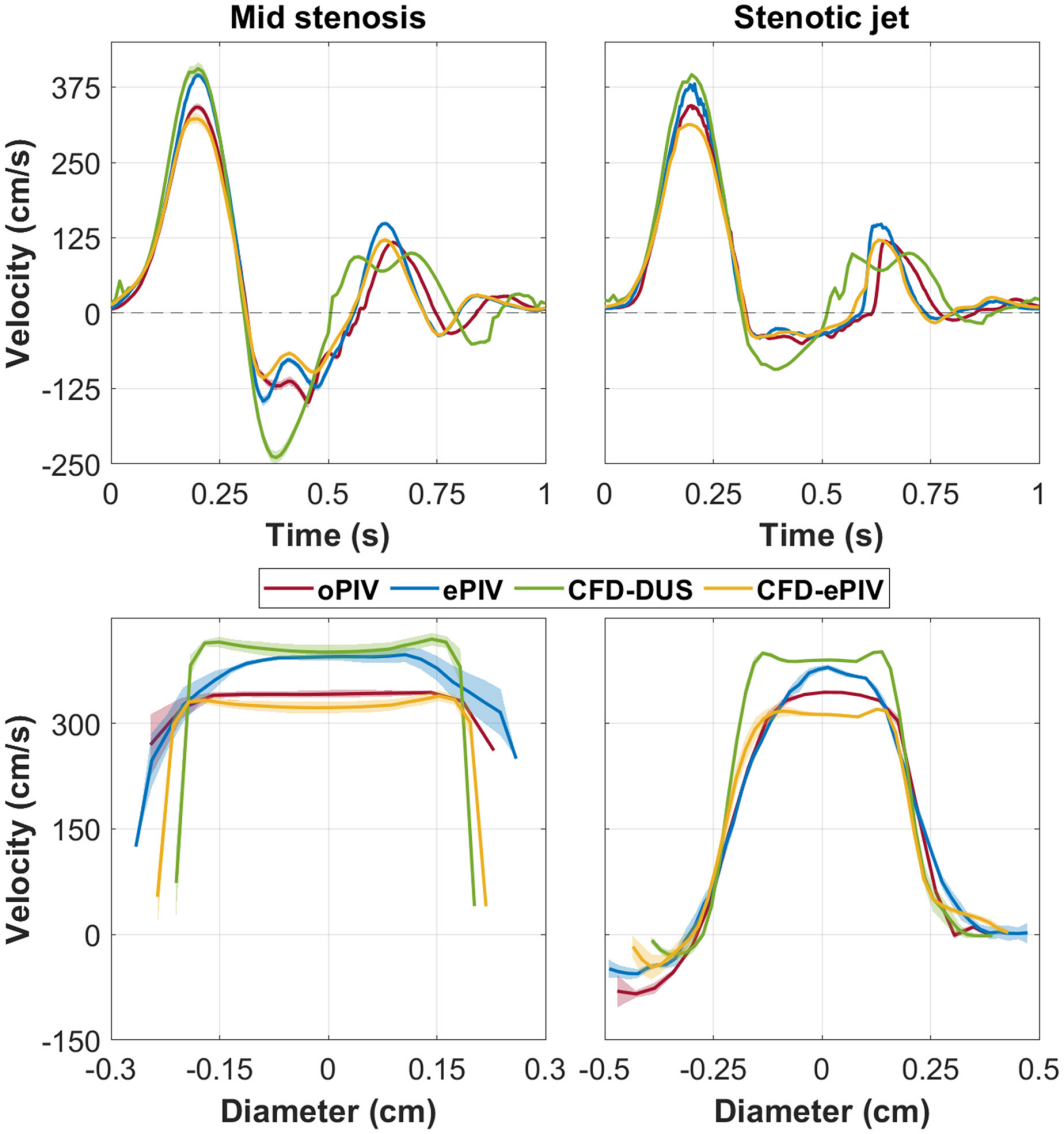
Temporal (left) and spatial (right) velocity profiles at mid stenosis and the post-stenotic jet in the stenosed model. Shading represents the standard deviation in space probed over five at a 0.5 mm distance of each other.

The spatial variation in the temporal and spatial velocity profiles at various points of interest are shown in Fig. 10 for the control model and Fig. 11 for the stenosed model.

## 10 Appendix E: Video Captions

The following videos are mentioned in the text:

Video 1, In-plane velocity vector fields over time obtained with all techniques in the control model (MPEG, 2.82 MB [URL: https://doi.org/10.1117/1.JMI.11.3.037001.s1]).
Video 2, In-plane velocity vector fields over time obtained with all techniques in the stenosis model (MPEG, 3.46 MB [URL: https://doi.org/10.1117/1.JMI.11.3.037001.s2]).

## Supplementary Material





## Data Availability

All data in support of the findings of this paper are available within the article or as supplementary material. Unprocessed data and codes used for analysis are available upon reasonable request to the corresponding author.

## References

[1] Song P. et al., “Global, regional, and national prevalence and risk factors for peripheral artery disease in 2015: an updated systematic review and analysis,” Lancet Glob. Heal. 7(8), E1020–E1030 (2019). 10.1016/S2214-109X(19)30255-4 31303293

[2] Wikström J. et al., “Lower extremity artery stenosis distribution in an unselected elderly population and its relation to a reduced ankle-brachial index,” J. Vasc. Surg. 50(2), 330–334 (2009). 10.1016/j.jvs.2009.03.008 19446989

[3] Gimbrone M. A. García-Cardeña G., “Vascular endothelium, hemodynamics, and the pathobiology of atherosclerosis,” Cardiovasc. Pathol. 22(1), 9–15 (2013). CATHE8 1054-8807 10.1016/j.carpath.2012.06.006 22818581 PMC4564111

[4] Holenstein R. Ku D. N., “Reverse flow in the major infrarenal vessels: a capacitive phenomenon,” Biorheology 25(6), 835–842 (1988). BRHLAU 0006-355X 10.3233/BIR-1988-25604 3076804

[5] Chi J. et al., “Assessment of femoral artery atherosclerosis at the adductor canal using 3D black-blood MRI,” Clin. Radiol. 68(4), 213–221 (2013). 10.1016/j.crad.2012.12.002 23332436

[6] Ambrogio S. et al., “Pulsed wave Doppler measurements of maximum velocity: dependence on sample volume size,” Ultrasound Med. Biol. 48(1), 68–77 (2022). USMBA3 0301-5629 10.1016/j.ultrasmedbio.2021.09.006 34607758

[7] Steinman A. H. et al., “Sources of error in maximum velocity estimation using linear phased-array Doppler systems with steady flow,” Ultrasound Med. Biol. 27(5), 655–664 (2001). USMBA3 0301-5629 10.1016/S0301-5629(01)00352-0 11397530

[r8] Ubbink D. T. Fidler M. Legemate D. A., “Interobserver variability in aortoiliac and femoropopliteal duplex scanning,” J. Vasc. Surg. 33(3), 540–545 (2001). 10.1067/mva.2001.111734 11241125

[9] Hussain S. T. Wood R. F. M. Bland M., “Observer variability in volumetric blood flow measurements in leg arteries using duplex ultrasound,” Ultrasound Med. Biol. 22(3), 287–291 (1996). USMBA3 0301-5629 10.1016/0301-5629(95)02050-0 8783460

[10] Stankovic Z. et al., “4D flow imaging with MRI,” Cardiovascular 4(2), 173–192 (2014). 10.3978/j.issn.2223-3652.2014.01.02 PMC3996243 24834414

[11] Frydrychowicz A. François C. J. Turski P. A., “Four-dimensional phase contrast magnetic resonance angiography: potential clinical applications,” Eur. J. Radiol. 80(1), 24–35 (2011). EJRADR 0720-048X 10.1016/j.ejrad.2011.01.094 21333479 PMC3116042

[12] Jensen J. A. et al., “Ultrasound vector flow imaging-Part I: sequential systems,” IEEE Trans. Ultrason. Ferroelectr. Freq. Control 63(11), 1704–1721 (2016). ITUCER 0885-3010 10.1109/TUFFC.2016.2600763 27824555

[13] Jensen J. A. et al., “Ultrasound vector flow imaging—Part II: parallel systems,” IEEE Trans. Ultrason. Ferroelectr. Freq. Control 63(11), 1722–1732 (2016). ITUCER 0885-3010 10.1109/TUFFC.2016.2598180 27824556

[14] Voorneveld J. et al., “High frame rate ultrasound particle image velocimetry for estimating high velocity flow patterns in the left ventricle,” IEEE Trans. Ultrason. Ferroelectr. Freq. Control 65(12), 2222–2232 (2018). ITUCER 0885-3010 10.1109/TUFFC.2017.2786340 29990263

[15] Zhang F. et al., “*In vitro* and preliminary *in vivo* validation of echo particle image velocimetry in carotid vascular imaging,” Ultrasound Med. Biol. 37(3), 450–464 (2011). USMBA3 0301-5629 10.1016/j.ultrasmedbio.2010.11.017 21316562 PMC3449315

[16] van Helvert M. et al., “High-frame-rate contrast-enhanced ultrasound particle image velocimetry in patients with a stented superficial femoral artery: a feasibility study,” Eur. Radiol. Exp. 6(1), 32 (2022). 10.1186/s41747-022-00278-w 35790584 PMC9256892

[17] Morris P. D. et al., “Computational fluid dynamics modelling in cardiovascular medicine,” Heart 102(1), 18–28 (2016). 10.1136/heartjnl-2015-308044 26512019 PMC4717410

[18] Taylor C. A. Fonte T. A. Min J. K., “Computational fluid dynamics applied to cardiac computed tomography for noninvasive quantification of fractional flow reserve: scientific basis,” J. Am. Coll. Cardiol. 61(22), 2233–2241 (2013). JACCDI 0735-1097 10.1016/j.jacc.2012.11.083 23562923

[19] Buchmann N. A. et al., “Particle image velocimetry (PIV) and computational fluid dynamics (CFD) modelling of carotid artery haemodynamics under steady flow: a validation study,” J. Biomech. Sci. Eng. 5(4), 421–436 (2010).1880-9863 10.1299/jbse.5.421

[20] Nørgaard B. L. et al., “Diagnostic performance of noninvasive fractional flow reserve derived from coronary computed tomography angiography in suspected coronary artery disease,” J. Am. Coll. Cardiol. 63(12), 1145–1155 (2014). JACCDI 0735-1097 10.1016/j.jacc.2013.11.043 24486266

[21] Varghese S. S. Frankel S. H. Fischer P. F., “Modeling transition to turbulence in eccentric stenotic flows,” J. Biomech. Eng. 130(1), 014503 (2008). JBENDY 0148-0731 10.1115/1.2800832 18298194

[22] Mancini V. et al., “High-frequency fluctuations in post-stenotic patient specific carotid stenosis fluid dynamics: a computational fluid dynamics strategy study,” Cardiovasc. Eng. Technol. 10(2), 277–298 (2019). 10.1007/s13239-019-00410-9 30937853 PMC6527791

[23] Kung E. et al., “*In vitro* validation of patient-specific hemodynamic simulations in coronary aneurysms caused by Kawasaki disease,” Cardiovasc. Eng. Technol. 5(2), 189–201 (2015). 10.1007/s13239-014-0184-8 PMC4103185 25050140

[24] Paliwal N. et al., “Methodology for computational fluid dynamic validation for medical use: application to intracranial aneurysm,” J. Biomech. Eng. 139(12) (2017). JBENDY 0148-0731 10.1115/1.4037792 PMC5686786 28857116

[25] Hansen K. L. et al., “Vector flow imaging compared with digital subtraction angiography for stenosis assessment in the superficial femoral artery: a study of vector concentration, velocity ratio and stenosis degree percentage,” Ultrasound Int. Open 5(2), E53–E59 (2019). 10.1055/a-0853-2002 30886943 PMC6420338

[26] Brindise M. C. Busse M. M. Vlachos P. P., “Density and viscosity matched Newtonian and non-Newtonian blood-analog solutions with PDMS refractive index,” Exp. Fluids 59, 173 (2018). EXFLDU 0723-4864 10.1007/s00348-018-2629-6 31745378 PMC6863344

[27] Thielicke W. Stamhuis E. J., “PIVlab: towards user-friendly, affordable and accurate digital particle image velocimetry in MATLAB,” J. Open Res. Software 2(1), e30 (2014). 10.5334/jors.bl

[28] Baranger J. et al., “Adaptive spatiotemporal SVD clutter filtering for ultrafast Doppler imaging using similarity of spatial singular vectors,” IEEE Trans. Med. Imaging 37(7), 1574–1586 (2018). ITMID4 0278-0062 10.1109/TMI.2018.2789499 29969408

[29] Voorneveld J. et al., “Optimization of microbubble concentration and acoustic pressure for left ventricular high frame rate EchoPIV in patients,” IEEE Trans. Ultrason. Ferroelectr. Freq. Control 68(7), 2432–2443 (2021). ITUCER 0885-3010 10.1109/TUFFC.2021.3066082 33720832

[30] Gerhard-Herman M. et al., “Guidelines for noninvasive vascular laboratory testing: a report from the American Society of Echocardiography and the Society for Vascular Medicine and Biology,” Vasc. Med. 11(3), 183–200 (2006). VMEREI 10.1177/1358863x06070516 17288127

[31] Updegrove A. et al., “SimVascular: an open source pipeline for cardiovascular simulation,” Ann. Biomed. Eng. 45(3), 525–541 (2017). ABMECF 0090-6964 10.1007/s10439-016-1762-8 27933407 PMC6546171

[32] Antiga L. et al., “An image-based modeling framework for patient-specific computational hemodynamics,” Med. Biol. Eng. Comput. 46(11), 1097–1112 (2008). MBECDY 0140-0118 10.1007/s11517-008-0420-1 19002516

[33] McGah P. M. et al., “*In vitro* validation of endovascular Doppler-derived flow rates in models of the cerebral circulation,” Physiol. Meas. 36(11), 2301–2317 (2015). PMEAE3 0967-3334 10.1088/0967-3334/36/11/2301 26450643 PMC4684705

[34] Bland J. M. Altman D. G., “A note on the use of the intraclass correlation coefficient in the evaluation of agreement between two methods of measurement,” Comput. Biol. Med. 20(5), 337–340 (1990). CBMDAW 0010-4825 10.1016/0010-4825(90)90013-F 2257734

[35] Batschelet E., Circular Statistics in Biology, Academic Press, London (1981).

[36] Pedersen M. M. et al., “Novel flow quantification of the carotid bulb and the common carotid artery with vector flow ultrasound,” Ultrasound Med. Biol. 40(11), 2700–2706 (2014). USMBA3 0301-5629 10.1016/j.ultrasmedbio.2014.06.001 25218449

[37] van de Velde L. et al., “Partial renal coverage in endovascular aneurysm repair causes unfavorable renal flow patterns in an infrarenal aneurysm model,” J. Vasc. Surg. 67(5), 1585–1594 (2018). 10.1016/j.jvs.2017.05.092 28893490

[38] Van Cauwenberge J. et al., “Assessing the performance of ultrafast vector flow imaging in the neonatal heart via multiphysics modeling and *in vitro* experiments,” IEEE Trans. Ultrason. Ferroelectr. Freq. Control 63(11), 1772–1785 (2016). ITUCER 0885-3010 10.1109/TUFFC.2016.2596804 27824560

[39] Hoskins P. R., “Estimation of blood velocity, volumetric flow and wall shear rate using Doppler ultrasound,” Ultrasound 19(3), 120–129 (2011). 10.1258/ult.2011.011015

[40] Raben J. S. et al., “Time-resolved particle image velocimetry measurements with wall shear stress and uncertainty quantification for the FDA Nozzle model,” Cardiovasc. Eng. Technol. 7(1), 7–22 (2016). 10.1007/s13239-015-0251-9 26628081

[41] Walsh M. et al., “On using experimentally estimated wall shear stresses to validate numerically predicted results,” Proc. Inst. Mech. Eng., Part H: J. Eng. Med. 217, 77–90 (2003). 10.1243/09544110360579286 12666774

[42] Leinan P. R. et al., “Comparison of ultrasound vector flow imaging and CFD simulations with PIV measurements of flow in a left ventricular outflow track phantom: implications for clinical use and *in silico* studies,” Comput. Biol. Med. 146, 105358 (2022). CBMDAW 0010-4825 10.1016/j.compbiomed.2022.105358 35751181

[43] Lan I. S. et al., “Validation of the reduced unified continuum formulation against in vitro 4D-flow MRI,” Ann. Biomed. Eng. 51(2), 377–393 (2023). ABMECF 0090-6964 10.1007/s10439-022-03038-4 35963921 PMC11402517

[44] Vos H. J. et al., “Contrast-enhanced high-frame-rate ultrasound imaging of flow patterns in cardiac chambers and deep vessels,” Ultrasound Med. Biol. 46(11), 2875–2890 (2020). USMBA3 0301-5629 10.1016/j.ultrasmedbio.2020.07.022 32843233

[45] Chnafa C. Mendez S. Nicoud F., “Image-based simulations show important flow fluctuations in a normal left ventricle: what could be the implications?” Ann. Biomed. Eng. 44(11), 3346–3358 (2016). ABMECF 0090-6964 10.1007/s10439-016-1614-6 27073110

[46] Voorneveld J. et al., “Native blood speckle vs ultrasound contrast agent for particle image velocimetry with ultrafast ultrasound: *in vitro* experiments,” in IEEE Int. Ultrasonics Symp. (IUS), pp. 1–4 (2016). 10.1109/ULTSYM.2016.7728614

[47] Colombo M. et al., “In-stent restenosis progression in human superficial femoral arteries: dynamics of lumen remodeling and impact of local hemodynamics,” Ann. Biomed. Eng. 49(9), 2349–2364 (2021). ABMECF 0090-6964 10.1007/s10439-021-02776-1 33928465 PMC8455500

[48] van de Velde L. et al., “Computational fluid dynamics for the prediction of endograft thrombosis in the superficial femoral artery,” J. Endovasc. Ther. 30(4), 615–627 (2022). 10.1177/15266028221091890 35466777 PMC10350734

[49] Hachem E. et al., “Reinforcement learning for patient-specific optimal stenting of intracranial aneurysms,” Sci. Rep. 13(1), 7147 (2023). SRCEC3 2045-2322 10.1038/s41598-023-34007-z 37130900 PMC10154322

[50] Colombo M. et al., “Superficial femoral artery stenting: impact of stent design and overlapping on the local hemodynamics,” Comput. Biol. Med. 143, 105248 (2022). CBMDAW 0010-4825 10.1016/j.compbiomed.2022.105248 35124437

[51] Engelhard S. et al., “US velocimetry in participants with aortoiliac occlusive disease,” Radiology 9, 210454 (2021). RADLAX 0033-8419 10.1148/radiol.2021210454 34427462

[52] Engelhard S. et al., “Blood flow quantification with high-frame-rate, contrast-enhanced ultrasound velocimetry in stented aortoiliac arteries: *in vivo* feasibility,” Ultrasound Med. Biol. 48, 1518–1527 (2022). USMBA3 0301-5629 10.1016/j.ultrasmedbio.2022.03.016 35577661

[53] Kaempf M. et al., “CT angiography of various superficial femoral artery stents: an in vitro phantom study,” Eur. J. Radiol. 81(7), 1584–1588 (2012). EJRADR 0720-048X 10.1016/j.ejrad.2011.04.014 21546182

[54] Sandgren T. et al., “The diameter of the common femoral artery in healthy human: influence of sex, age, and body size,” J. Vasc. Surg. 29(3), 503–510 (1999). 10.1016/S0741-5214(99)70279-X 10069915

[55] Zierler R. E. Zierler B. K., “Duplex sonography of lower extremity arteries,” Semin. Ultrasound CT MRI 18(1), 39–56 (1997). 10.1016/S0887-2171(97)90037-8 9143065

[56] Wood N. B. et al., “Curvature and tortuosity of the superficial femoral artery: a possible risk factor for peripheral arterial disease,” J. Appl. Physiol. 101, 1412–1418 (2006). 10.1152/japplphysiol.00051.2006 16825527

[57] Bishop P. D. et al., “PVSS9. Geometric and morphological analyses of the superficial femoral artery by IVUS,” J. Vasc. Surg. 51(6), 51S–52S (2010). 10.1016/j.jvs.2010.02.143

[58] Sur Y. J. et al., “The first perforating branch of the deep femoral artery: a reliable recipient vessel for vascularized fibular grafts: an anatomical study,” J. Plast. Reconstr. Aesthetic Surg. 69(3), 351–358 (2016). 10.1016/j.bjps.2015.10.024 26626196

[59] Dixit D. et al., “A study of variations in the origin of profunda femoris artery and its circumflex,” Int. J. Biol. Med. Res. 2(4), 1084–1089 (2011).

